# Model-Free Cluster Analysis of Physical Property Data using Information Maximizing Self-Argument Training

**DOI:** 10.1038/s41598-020-64281-0

**Published:** 2020-05-13

**Authors:** Ryohto Sawada, Yuma Iwasaki, Masahiko Ishida

**Affiliations:** 1 System Platform Research Laboratories, NEC Corporation, Tsukuba, 305-8501 Japan; 2 PRESTO, JST, Saitama, 322-0012 Japan

**Keywords:** Computational methods, Computer science

## Abstract

We present semi-supervised information maximizing self-argument training (IMSAT), a neural network-based classification method that works without the preparation of labeled data. Semi-supervised IMSAT can amplify specific differences and avoid undesirable misclassification in accordance with the purpose. We demonstrate that semi-supervised IMSAT has a comparable performance with existing methods for semi-supervised learning of image classification and can also classify real experimental data (X-ray diffraction patterns and thermoelectric hysteresis curves) in the same way even though their shape and dimensions are different. Our algorithm will contribute to the automation of big data processing and artificial intelligence-driven material development.

## Introduction

High-throughput materials fabrication and characterization are in strong demand in the field of material development due to the increasing complexity of the industrial materials^1,2^. The composition-spread technique is a promising solution where one can fabricate the gradient of a composition in a single fabrication. For example, Yoo *et al*., fabricated a Fe-Ni-Co ternary alloy and measured a continuous phase diagram^3^ and Wang *et al*., fabricated La_1−*x*_(Ca, RE)_*x*_VO_3_ composition-spread films and measured thermoelectricity^4^. Furthermore, high-throughput materials fabrication also enables to apply big data analysis to material development. Big data analysis helps to discover unexpected features and new materials^5–7^.

High-throughput data processing is inevitable to utilize high-throughput material fabrication. However, the automation of the data processing is challenging for two reasons. First, raw experimental data varies depending on not only essential physical properties but also unessential experimental conditions. Second, one usually needs to classify the data based on purpose-specific rules. For example, for X-ray diffraction (XRD), the spectrum varies depending on not only the crystal structure but also experimental conditions such as the power of the source, sensitivity of the detector, and background noise^8^. The dependence on the experimental conditions not only makes analysis costly but also prevents data sharing between different databases. Additionally, a noteworthy feature of the spectrum changes depending on the purpose. For example, one needs to focus on the position of the peak to classify the crystal structure. On the other hand, to evaluate the purity of the crystal, one needs to focus on the width of the peak^9^. For these reasons, to realize automatic classification, an algorithm that enables users to adjust the classification method while working with a small amount of data is required.

A machine-learning approach is a good solution if there is a sufficient amount of labeled data. A neural network is especially promising because it can handle various types of data^10^. Neural network can solve various problems without domain knowledge (e.g. image recognition, text recognition and sound recognition^11,12^, crystal structure^13^ chaotic phase and quantum mechanics^14–16^). However, a neural network requires a large amount of labeled data for supervised learning, and data collection is difficult in real experimental data.

In terms of profit, it is desirable to use unsupervised learning that does not require labeled data. The key question for automated classification using unsupervised learning is how to quantify the similarity between two pieces of data. For XRD, the spectrum is given as $s(x)$ where $x$ is the diffraction angle. The similarity between the two pieces of data $s,t$ is defined by the kernel function $D(s,t)$. Iwasaki *et al*. tried to classify the XRD data of Fe-Co-Ni ternary-alloy thin film by using several kernel functions (Euclidean, Manhattan, Pearson, cosine, and normalized and constrained dynamic time warping (NC-DTW)) and found only NC-DTW can classify a crystal structure because it can accommodate peak shifting due to lattice constant change^17^. In NC-DTW, $D(s,t)$ is given by

1 $$D(s,t)=\,{\rm{\min }}\,\mathop{\sum }\limits_{i}^{N}\,{({s{\prime} }_{j(i)}-{t{\prime} }_{i})}^{2}$$

where

2 $$s{\prime} =\frac{s}{{({\sum }_{i}^{N}{s}_{i}^{2})}^{1/2}},t{\prime} =\frac{s}{{({\sum }_{i}^{N}{t}_{i}^{2})}^{1/2}}$$

and *j*(*i*) must satisfy

3 $$\begin{array}{ll}i\le i{\prime} \Rightarrow j(i)\le j(i{\prime} ), & |j(i)-i| < w,\\ j(1)=1,\,j(N)=N.\end{array}$$

where $w$ is the window size that limits the range of time warping. However, the appropriate kernel function varies depending on the problem. For XRD, NC-DTW is suitable only because the XRD spectrum can move depending on the lattice constant. Furthermore, many of the existing kernel functions, including NC-DTW, are limited to low dimensional classification, even though a lot of raw experimental data is complicated multi-dimensional data. These problems prevent us from reusing kernel functions and make the automation non-profitable.

In this paper, we present a comprehensive solution based on information maximizing self-argument training (IMSAT)^18^ that uses a neural network to maintain versatility and does not require manual kernel function searches or preparation of labeled data. We demonstrate our algorithm performs comparably with existing methods for semi-supervised learning of image classification and succeeds in classifying line charts and scatter plots from raw experimental data. Our algorithm can accelerate the automation of big data collection and open the way to the study of artificial intelligence-driven material development.

### Semi-supervised IMSAT

Model complexity is the core of a neural network’s versatility; however, it is also the reason that a neural network can easily overfit small data sets. Therefore, the degree of freedom of the neural network needs to be reduced to avoid overfitting by “regularization”. Recently, the neural network regularized by Virtual Adversarial Training (VAT) succeeded in clustering handwritten numerals with only a small amount of data. VAT^19^ is a representative regularization method based on local perturbation. The objective function of VAT is defined by the following function:

4 $${R}_{vat}(\theta )={R}_{pert}(\theta )+{H}_{l}(\theta )$$

where

$$\begin{array}{rcl}{R}_{pert}(\theta ) & = & \mathop{\sum }\limits_{i}^{N}\,(\,-\,\mathop{\sum }\limits_{y{\prime} }^{{V}_{y}}\,{p}_{\theta }(y{\prime} |{x}_{i})\,{\log }_{{p}_{\theta }}\,(y{\prime} |{T}_{\theta }({x}_{i})))\\ {H}_{l}(\theta ) & = & \beta (\mathop{\sum }\limits_{j}^{{N}^{l}}\,\log \,{p}_{\theta }({y}_{j}^{l}|{x}_{j}^{l})),\end{array}$$

*θ* is parameter of the neural network, $N$ is the number of data, ${x}_{i}$ is the $i$-th data, ${V}_{y}$ is the number of clusters, $p(y|x)$ is conditional probability, ${T}_{\theta }({x}_{i})$ is the perturbated data, ${N}_{l}$ is the number of data with label information, and $\beta$ is a hyper parameter. ${H}_{l}$ is the same as the target function of supervised learning. ${T}_{\theta }({x}_{i})$ is chosen to be

5 $$\begin{array}{rcl}{T}_{\theta }(x) & = & {\rm{\arg }}\,\mathop{{\rm{\max }}}\limits_{x{\prime} }\,{R}_{vat}(\theta ;x,x{\prime} )\\  & = & {\rm{\arg }}\,\mathop{{\rm{\max }}}\limits_{x{\prime} }-\mathop{\sum }\limits_{y{\prime} }^{{V}_{y}}\,{p}_{\theta }(y{\prime} |{x}_{i})\,{\log }_{{p}_{\theta }}\,(y{\prime} |x{\prime} ).\end{array}$$

Regularization using local perturbation is based on the idea that it is preferable for data representations to be locally invariant (i.e., remain unchanged under local perturbations on data points). The idea would enable neural networks to learn meaningful representations of data.

IMSAT is an expansion of VAT for unsupervised learning. The objective function of IMSAT is defined by the following equation:

6 $${R}_{pert}(\theta )-\lambda (\mu H(y)-H(y|x))$$

where $\mu$ and $\lambda$ are hyper parameters, $H(y)$ and $H(y|x)$ are marginal entropy and conditional entropy, respectively,

7 $$H(y)=h(\frac{1}{N}(\mathop{\sum }\limits_{i}^{N}\,{p}_{\theta }(y|{x}_{i}))$$

8 $$H(y|x)=\frac{1}{N}\,\mathop{\sum }\limits_{i}^{N}\,h({p}_{\theta }(y|{x}_{i}))$$

and $h({p}_{\theta }(y|x))$ is the entropy function

9 $$h({p}_{\theta }(y))=-\,\sum _{y{\prime} }\,{p}_{\theta }(y{\prime} )\,\log \,({p}_{\theta }(y{\prime} )).$$

Increasing the marginal entropy $H(y)$ encourages uniformity among the cluster sizes, while decreasing the conditional entropy $H(y|x)$ encourages unambiguous cluster assignments. IMSAT achieved over 90% accuracy in unsupervised learning of the clustering of handwritten numerals.

The original IMSAT is not suitable for regarding specific differences as important because IMSAT only attempts to make data representation locally invariant. However, specific differences are sometimes regarded as important due to domain knowledge. Therefore, we added ${H}_{I}$ to enable semi-supervised learning. Our algorithm optimizes the following function:

10 $${R}_{vat}(\theta )-\lambda (\mu H(y)-H(y|x)).$$

Semi-supervised IMSAT has two advantages in terms of the application to real experimental data. The first is it can amplify specific differences and modify the classification method in accordance with the purpose. The second is it does not restrict data structures. Many current semi-supervised learning methods use data-structure dependent augmentations such as flipping, rotation, and color filtering to improve accuracy. On the other hand, semi-supervised IMSAT is applicable to most of the existing network architectures without restricting data structure.

## Results

### Comparison with existing algorithms

We compared the classification accuracies of VAT, IMSAT, semi-supervised IMSAT (our method) and mean teacher^20^ for handwritten digit images (MNIST) download from^21^. We addressed two tasks, usual classification, and classification using a quotient divided by two where $[0,1]$, $[2,3]$, $[4,5]$, $[6,7]$, $[8,9]$ are classified as the same group respectively. We used 64 images for labeled training data, 10,000 images for testing, and 60,000 images for unlabeled data for semi-supervised learning. Table 1 shows the classification results. Semi-supervised IMSAT outperforms VAT, IMSAT, and mean teacher in classifying the quotients. This indicates that semi-supervised IMSAT is suitable for modifying the classification method in accordance with a user-specific purpose.

**Table 1:** Classification accuracies of VAT, IMSAT, semi-supervised IMSAT (our method) and mean teacher for handwritten digit images (MNIST).

	VAT	IMSAT	our method	mean teacher
Normal	96.3%	95.8%	96.1%	93.6%
Quotient	72.5%	48.2%	93.7%	90.5%

### Clustering line chart (XRD patterns)

**Figure 1:**
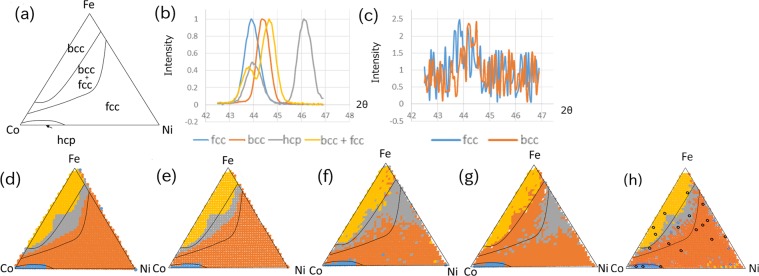
Result of clustering of XRD data of Fe-Co-Ni ternary-alloy thin film. (**a**) Phase map manually deduced from individual XRD patterns of spread wafer. (**b**) Example of XRD patterns where random noise was added to the diffraction data. (**c**) Example of XRD patterns where random noise was added. (**d**) Result of cluster analysis using IMSAT (${V}_{y}=4$) and (**e**) that using NC-DTW. (**f**) Result of cluster analysis using IMSAT (${V}_{y}=4$), (**g**) that using NC-DTW, (**h**) and that using semi-supervised IMSAT, where random noise was added to the diffraction data. We used 16 labeled data for semi-supervised IMSAT(shown by dots).

We applied our algorithm to the clustering of a line chart. Figure 1(a) shows the phase map manually deduced from individual XRD patterns of a Fe-Co-Ni ternary-alloy thin film^17^. The XRD patterns are from ref. ^3^. The number of data $N$ is 1240. There are four types of diffraction data, fcc (face centered cubic), bcc (body centered cubic), hcp (hexiagonal closed packed), and combination of fcc and bcc^8,9^. Examples of XRD patterns are shown in Fig. 1(b). The automated composition-phase maps identified using IMSAT and NC-DTW are shown in Fig. 1(d,e), respectively. These maps appear to be nearly the same.

We also examined how robust these algorithms are to the noise in the data. Figure 1(c) shows examples of XRD patterns where random noise was added to the diffraction data. The XRD patterns are noisy and difficult to manually classify. Figure 1(f–h) show the automated composition-phase maps identified using IMSAT, NC-DTW and semi-supervised IMSAT, respectively. Surprisingly, IMSAT succeeded in clustering noisy XRD patterns and was more accurate than NC-DTW. Additionally, misclassification of bcc + fcc area was corrected by semi-supervised learning.

### Clustering scatter graph (hysteresis curve)

**Figure 2:**
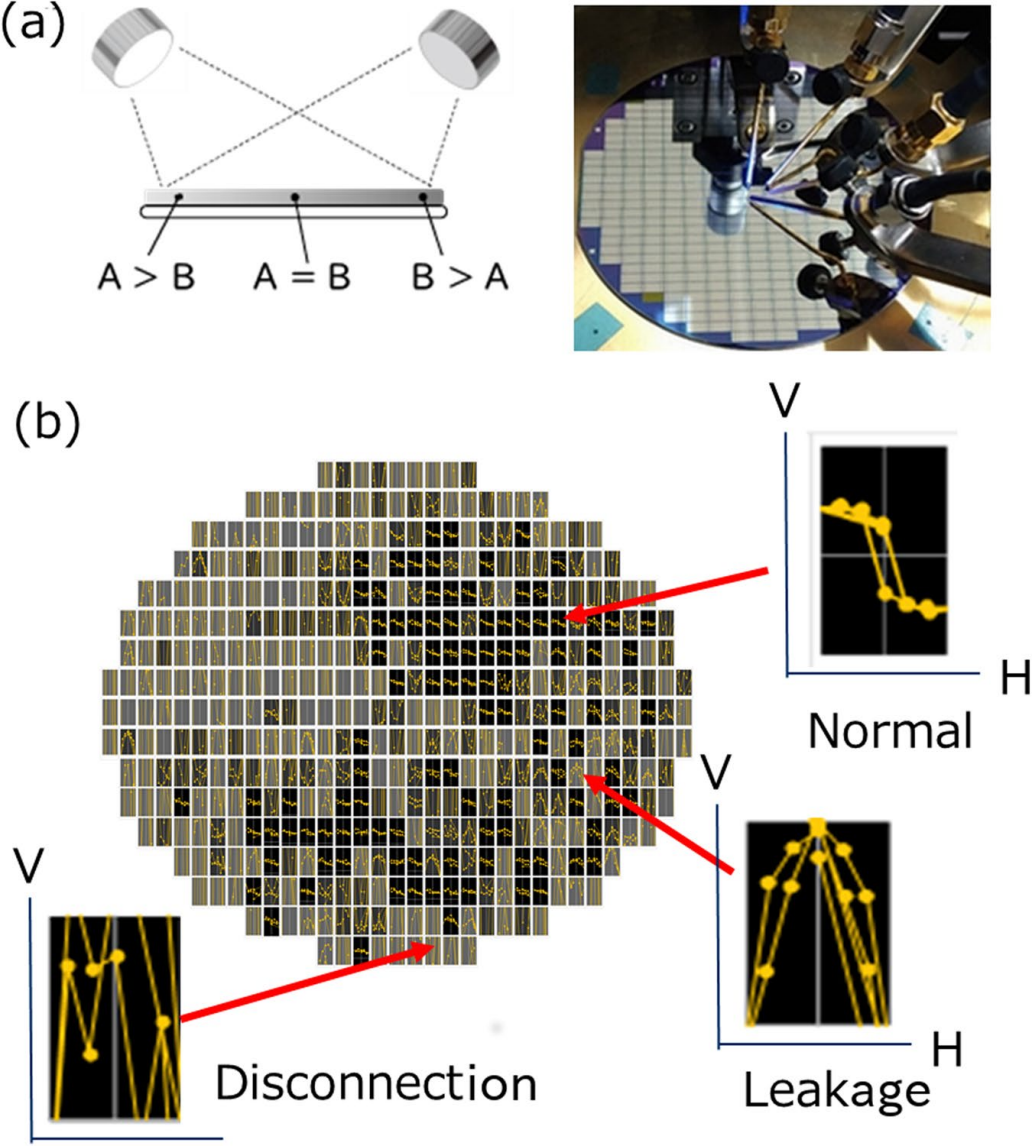
(**a**) Magnetic thin film fabricated by composition-spread sputtering. (**b**) Hysteresis curve of ANE exhibited by thermo electric voltage depending on the external magnetic field and examples of the thermoelectric voltage curve of the disconnected and leaked samples (**b**). We measured the thermoelectric voltages of the thin film using a semi-automatic wafer prober^24^.

To verify the versatility, we also applied IMSAT to the clustering of scatter graph data; clustering of the hysteresis curve of a magnetic FePt thin film. The FePt thin film was fabricated by composition-spread sputtering. Figure 2 shows an example of the thin film fabricated by composition-spread sputtering (a) and the hysteresis curve of the anomalous Nernst effect (ANE) where thermo electric voltage exhibits a hysteresis curve depending on the external magnetic field (b)^22,23^. The shape of the curve will change if fabrication of the thin film fails. There are two reasons for failure, disconnection inside the sample and the insulator basis leaking onto the sample. Figure 2(b) shows examples of the thermoelectric voltage curve of the disconnected and leaked samples. Typical curves of the disconnected and leaked samples are random noise and a V-shaped curve, respectively.

The left column of Table 2 shows the automatic clustering results of the FePt thin film’s ANE voltage curve using IMSAT. Manual clustering was implemented by considering the curvature shape and the results of the four-terminal measurement. Clearly, our algorithm was successful and highly accurate in classifying the normal samples. However, the classification accuracy of the disconnected and leaked samples was not so high, possibly because disconnection and leakage can occur simultaneously.

**Table 2:** Result of automatic clustering of the voltage curve of ANE of FePt thin film using IMSAT and semi-supervised IMSAT.

	Results with unsupervised IMSAT			Results with semi-supervised IMSAT		
	Normal	Disconnect	Leak	Normal	Disconnect	Leak
Normal (manual)	95	0	2	95	1	1
Disconnect (manual)	18	165	95	4	239	35
Leak (manual)	13	12	28	4	18	31
Recall	0.944	0.595	0.528	0.944	0.823	0.570
Precision	0.753	0.932	0.224	0.922	0.926	0.462
${R}_{pert}$	0.275			0.465		

In terms of industrialization, classifying a failed sample as a normal sample is critical. The left column of Table 2 shows that IMSAT sometimes classified a failed sample as a normal sample because IMSAT only attempts to make data representation locally invariant. We addressed the problem with semi-supervised learning where a penalty is added to the misclassification of labeled data. The samples for labeled data are randomly chosen from those that are classified as normal by IMSAT even though they were manually classified as failed samples. We set ${N}_{l}$ as $5$ and $\beta$ as $3.34$. The right column of Table 2 shows the result of automatic clustering using semi-supervised learning. Semi-supervised learning suppressed the misclassification by adding a penalty, but it increased ${R}_{pert}$ at the same time. This indicates semi-supervised IMSAT can flexibly respond to a user’s needs by regarding small, specific differences as important. We could not achieve 100% accuracy with a normal sample, possibly because the amounts of disconnection and leakage were not discrete quantities.

## Discussion

We presented how semi-supervised IMSAT can effectively classify raw experimental data without manual kernel function searches or preparation of large amounts of labeled data. We demonstrated semi-supervised IMSAT performs comparably with existing algorithms in the clustering of handwritten digits. We also applied semi-supervised IMSAT to the clustering of XRD patterns and the thermoelectric curve and showed that semi-supervised IMSAT is versatile and robust against noise and easily tunable by small data. Our algorithm can accelerate the automation of big data collection and open the way to the study of artificial intelligence-driven material development.

## Methods

### Condition for the clustering

We used 3-layer convolutional neural network for the clustering by mean teacher with kernel size 5. We optimized consistency weight to 1.0 to maximize the accuracy.

We used commonly reported parameter values for the clustering by VAT, IMSAT and semi-supervised IMSAT. We set the network dimensionality to *d*-1200-1200-*V*_*y*_ for the clustering of XRD patterns, where *d*(=89) is input dimensionality. ${N}_{l}$, $\mu$, and $\lambda$ were set to 0 (unsupervised learning), $0.2$, and $0.2$, respectively. We set the size of the mini-batch to 64 and ran 50 epochs. We also tried the clustering using NC-DTW. We used the same parameters as Iwasaki’s paper for NC-DTW. We set the window size $w$ to be 10 (0.5 degrees) and used hierarchy clustering analysis with the average linkage method.

The parameter values for neural networks for the clustering of the ANE voltage curve were almost the same as the clustering of XRD patterns. We set the network dimensionality to *d*-1200-1200-*V*_*y*_ for the clustering, where *d*(=28 × 28) is input dimensionality. ${N}_{l}$, $\mu$, and $\lambda$ were set to be 0 (unsupervised learning), 0.2, and 0.2, respectively. We set the size of the mini-batch to 40 and ran 50 epochs.

## Data Availability

The datasets generated and analyzed during the current study are available from the corresponding author on reasonable request.
